# Immunogenicity and Protective Efficacy of a Vero Cell Culture-Derived Whole-Virus H7N9 Vaccine in Mice and Guinea Pigs

**DOI:** 10.1371/journal.pone.0113963

**Published:** 2015-02-26

**Authors:** Walter Wodal, Michael G. Schwendinger, Helga Savidis-Dacho, Brian A. Crowe, Christine Hohenadl, Richard Fritz, Peter Brühl, Daniel Portsmouth, Anita Karner-Pichl, Dalida Balta, Leopold Grillberger, Otfried Kistner, P. Noel Barrett, M. Keith Howard

**Affiliations:** 1 Vaccine R&D, Baxter BioScience, Orth/Donau, Austria; 2 Pharmacology R&D, Baxter BioScience, Orth/Donau, Austria; 3 Process Development R&D, Baxter BioScience, Orth/Donau, Austria; University of Georgia, UNITED STATES

## Abstract

**Background:**

A novel avian H7N9 virus with a high case fatality rate in humans emerged in China in 2013. We evaluated the immunogenicity and protective efficacy of a candidate Vero cell culture-derived whole-virus H7N9 vaccine in small animal models.

**Methods:**

Antibody responses induced in immunized DBA/2J mice and guinea pigs were evaluated by hemagglutination inhibition (HI), microneutralization (MN), and neuraminidase inhibition (NAi) assays. T-helper cell responses and IgG subclass responses in mice were analyzed by ELISPOT and ELISA, respectively. Vaccine efficacy against lethal challenge with wild-type H7N9 virus was evaluated in immunized mice. H7N9-specific antibody responses induced in mice and guinea pigs were compared to those induced by a licensed whole-virus pandemic H1N1 (H1N1pdm09) vaccine.

**Results:**

The whole-virus H7N9 vaccine induced dose-dependent H7N9-specific HI, MN and NAi antibodies in mice and guinea pigs. Evaluation of T-helper cell responses and IgG subclasses indicated the induction of a balanced Th1/Th2 response. Immunized mice were protected against lethal H7N9 challenge in a dose-dependent manner. H7N9 and H1N1pdm09 vaccines were similarly immunogenic.

**Conclusions:**

The induction of H7N9-specific antibody and T cell responses and protection against lethal challenge suggest that the Vero cell culture-derived whole-virus vaccine would provide an effective intervention against the H7N9 virus.

## Introduction

A novel influenza A/H7N9 virus emerged in February in China in 2013 [1–3] which infects humans and causes severe lower respiratory tract infections, with clinical symptoms including pneumonia, respiratory failure, acute respiratory distress syndrome (ARDS) and multiorgan failure [1,4]. Despite the fact that most patients were treated in intensive care units [4,5], human H7N9 infections have resulted in a case fatality rate of approximately 30%. More than 400 cases have been reported in mainland China, Taiwan and Hong Kong, the majority in a second wave of infections in 2014. Several family clusters of H7N9 infection and one case of probable human to human transmission have been documented [5,6], but sustained transmission between humans has not yet occurred. However, a number of H7N9 features cause concern that this virus might readily adapt to more efficient transmission between humans.

The novel H7N9 virus binds both to avian (α2,3-linked sialic acid) and human (α2,6-linked sialic acid) receptors [7–9], can invade epithelial cells in the human lower respiratory tract [9] and type II pneumonocytes in alveoli [9], and replicates efficiently in *ex vivo* lung and trachea explant cultures [9,10]. H7N9 virus isolated from humans has been shown to replicate in human lung tissue as efficiently as seasonal influenza virus [11], associated with the potent ability of the H7N9 NS1 protein to inhibit the human antiviral IFN response [11]. Moreover, H7N9 attaches to epithelium in both the upper and lower human respiratory tract, a pattern which has not previously been reported for any avian influenza virus [12]. H7N9 isolates have also been shown to replicate efficiently in the upper and lower respiratory tracts of nonhuman primates [13], and limited transmission by respiratory droplets between ferrets has been demonstrated [13,14]. Several H7N9 isolates were also shown to contain amino acid changes which facilitate infection of mammals [13], and to contain a deletion in the NA stalk similar to an NA stalk deletion in H5N1 viruses which facilitates virus replication in the respiratory tract, and which might also be associated with adaptation and transmission in domestic poultry [1].

Before the emergence of the novel H7N9 virus, transmission of H7 viruses from birds to mammals had been reported only rarely, and human infections with N9 subtype viruses had not been reported. Accordingly, in a seroepidemiological study, no pre-existing immunity to H7N9 was detected in any age groups [9], and no detectable cross-reactive antibodies against the H7N9 virus were induced by immunization with a seasonal influenza vaccine [9]. If the novel H7N9 virus acquires the ability to transmit efficiently between humans, a safe and effective H7N9 vaccine will thus be urgently required. In the present study we investigated the immunogenicity of a Vero cell culture-derived whole-virus H7N9 vaccine in guinea pigs and mice. Antibody responses to both HA and NA were assessed, and the ability of the vaccine to protect mice against lethal challenge with wild-type H7N9 virus was evaluated. T-helper cell responses induced in immunized mice were analyzed by IFN-γ and IL-4 ELISPOT, and HA-specific IgG subtype analysis was done by ELISA. To investigate a hypothesis that H7N9 vaccines would be less immunogenic than other influenza vaccines [15], we compared the immunogenicity of the novel H7N9 vaccine to a licensed H1N1 (H1N1pdm09) vaccine [16–18] which was widely used in the 2009–2010 pandemic [19].

## Materials and Methods

### Ethics statement

All animal experiments were reviewed and approved by the Baxter Bioscience Institutional Animal Care and Use Committee (IACUC Vienna/ Orth) and animal welfare officers. Animal experiments were conducted in accordance with Austrian laws on animal experimentation and approved by Austrian regulatory authorities (permit number LF1 TVG-38/009–2011). Experiments were conducted according to guidelines set out by the Association for Assessment and Accreditation of Laboratory Animal Care International (AAALAC). Animals were housed according to EU guidelines, in housing facilities accredited by the AAALAC, and monitored daily. For blood sampling and virus challenge, all animals were anaesthetized with isoflurane using a UNO—Univentor Anaesthesia Unit according to the manufacturer`s protocol. Humane endpoints were used during the study, based on weight loss—animals with >25% weight loss were sacrificed using carbon dioxide treatment. All efforts were made to minimize suffering.

### Vaccine and viruses

The non-adjuvanted Vero cell culture-derived inactivated whole-virus H7N9 vaccine was manufactured using a H7N9 A/Anhui/1/2013 virus (E3;35590;13/104) obtained from the National Institute for Biological Standards and Control, UK. Vaccine virus was grown in Vero cell culture and double inactivated with formalin and UV irradiation, then purified by continuous sucrose gradient centrifugation and ultra/diafiltration steps prior to formulation, as previously described for licensed H1N1pdm09 and H5N1 vaccines [17,20].

### Immunization and challenge

Groups of 10 female DBA/2J mice (Janvier), aged 6–9 weeks, or groups of 5 female Duncan-Hartley guinea pigs (Charles River Laboratories, Germany), aged 6–9 weeks, were injected subcutaneously twice, three weeks apart, with six different 5-fold dilutions of vaccine ranging from 3.75 μg to 0.0012 μg HA antigen (in a volume of 0.5 mL) or buffer. For the H7N9 vaccine, due to the non-availability of standardized reagents, HA antigen content was estimated based on total protein content, whereby 10 μg total protein is estimated to contain 3.75 μg HA antigen, based on the HA content of the H1N1pdm09 comparator vaccine, as measured by SRD analysis. Additional characterization of the content of the H7N9 and H1N1pdm09 viral proteins HA, NA, NP and M1 was carried out by SDS gel analysis followed by densitometric analysis of coomassie-stained SDS-PAGE gels. Individual bands were identified by excision and in-gel digested with trypsin. Tryptic peptides were then analyzed by liquid chromatography-mass spectroscopy and the masses of the peptides searched against virus specific protein databases (GISAID EpiFlu database “h7n9_anhui_all.fasta” for H7N9, or “uniprot_sprot_090210_IAA_TR.fasta” and “A_California_07_2009_H1N1.fasta” for H1N1pdm09) using the search engine “BioworksBrowser”. Densitometry measurements were calibrated against a purified GAPDH protein standard. Viral RNA content was determined by extracting RNA from vaccine preparations using hot guanidinium thiocyanate-phenol-chloroform-based extraction method (TriReagent BD, Sigma Aldrich), followed by analysis using the Agilent Small RNA Kit and Agilent Bioanalyzer 2100 (Agilent Biotechnologies), according to the manufacturer’s instructions. Blood was drawn three weeks after the second immunization for serological analyses. In addition, three weeks after the second immunization with H7N9 vaccine, mice were challenged intranasally with 1 x 10^5^ TCID_50_ (5000 LD_50_) of H7N9 virus in 50 μl buffer (25 μl in each nostril). Challenged mice were monitored each work day for 14 days after challenge. Clinical symptoms were categorised as ‘ruffled fur’, ‘hunched back’, or ‘lethargic’; each category being assigned a numerical value of 1, and scored cumulatively for individual mice. Animals dying within the 14 day observation period were assigned a value of 6. For T cell analyses and IgG subclass determination in H7N9-immunized mice, blood was drawn and spleens were harvested from 5 of 10 mice 6 days after the first immunization and from the remaining 5 mice three weeks after the second immunization. Animal experiments were conducted in accordance with Austrian laws and guidelines set out by the Association for Assessment and Accreditation of Laboratory Animal Care International.

### Serological assays

Blood was drawn 3 weeks after the second immunization for serological analyses. HI antibody titers were determined using chicken or horse erythrocytes as previously described [17]. Sera with a negative signal at the first dilution (1:10) were assigned a nominal HI titer of 5. Virus-neutralizing antibodies were detected using a CPE-based microneutralization (MN) assay, essentially as previously described [20]. NAi antibodies were detected using an enzyme-linked lectin assay, essentially as previously described [21], using a recombinant A/Anhui/1/2013 (H7N9) N9 NA (Sino Biological Inc.; Cat. no. 40108-VNAHC; Lot no. LC07AP2401) or a recombinant A/California/04/2009 (H1N1pdm09) N1 NA (Sino Biological Inc.; Cat. no. 1158-VNAHC; Lot no. LC04NO1601). The NAi titer of a sample was defined as the 50% inhibiting titer.

### IFN-γ and IL-4 ELISPOT assay

The frequency of H7N9-specific IFN-γ- or Interleukin-4 (IL-4)-secreting cells was analyzed using mouse IFN-γ and IL-4 enzyme-linked immunospot (ELISPOT) kits (Mabtech AB, Nacka, Sweden), as previously described [20], using vaccine antigen at a concentration of 0.3 μg HA/ml.

### IgG subclass determination

ELISA plates were coated overnight at 4°C with either recombinant H7N9 HA (rHA) or polyclonal anti-mouse Fab2 IgG (Sigma). After blocking non-specific binding and subsequent washing, diluted sera or serial dilutions of purified murine IgG1, IgG2a or IgG2b (Sigma) were added to wells containing H7N9 rHA (Sino Biological Inc. Cat. no. 40103-V08B) or Fab2 IgG. Plates were incubated for 1 h at room temperature, and washed again, prior to further incubation for 1 h with IgG subclass-specific peroxidase-conjugated goat anti-mouse IgG antibodies. Bound IgG subclass antibodies were detected colorimetrically using TMB substrate.

### Statistical analyses

ED_50_ (the vaccine dose required to induce titer increases in 50% of immunized animals) and PD_50_ (the vaccine dose required to protect 50% of immunized animals against lethal challenge with wild-type H7N9 virus) values were calculated using in-house software based on the one-hit model [22]. Statistical relationships between vaccine-induced antibody titers, and the correlation of antibody titers with protection, were calculated using Spearman’s rank correlation coefficient (GraphPad Prism software, version 5). The significance of dose responses was assessed by one-way ANOVA and Kruskal-Wallis test (GraphPad). Comparison of antibody titers and T cell responses was done by two-way ANOVA and Bonferroni posttest (GraphPad). Comparison of IgG subclass responses was done by ANOVA with Kruskal-Wallis test using Dunn’s multiple comparison.

## Results

### Vaccine characterization

Results of the characterization of the content of the H7N9 and H1N1pdm09 viral proteins by SDS gel and densitometric analyses of coomassie-stained SDS-PAGE gels are shown in Table 1. Importantly, the content of HA proteins in the two vaccines is almost identical (50% and 30% of the viral and total protein content, respectively for the H7N9 vaccine compared to 53% and 30% for the H1N1pdm09 vaccine). The content of the NA, NP and M1 viral proteins is also highly similar in the two vaccines (Table 1). The viral RNA content of the H7N9 vaccine was 130±9 pg/μl, compared to 382±133 pg/μl for the H1N1pdm09 vaccine.

**Table 1:** % Viral and Total Protein ratios of H1N1pdm09 and H7N9 vaccines^a^.

	Viral protein ratio (%)		Total protein ratio (%)	
	H1N1pdm09	H7N9	H1N1pdm09	H7N9
HA	53	50	31	31
NA	16	23	10	14
NP	9	11	5	7
M1	23	16	13	10

^a^analysis based on the major analyzed bands HA1 + HA2, NA, NP and M1

### Dose-dependent induction of HI, MN and NAi antibodies in guinea pigs and mice

The ability of the whole-virus H7N9 vaccine to elicit H7N9-specific HI, MN and NAi antibodies was investigated by immunization of guinea pigs and DBA/2J mice. To investigate the hypothesis that H7N9 vaccines would be less immunogenic than other influenza vaccines [15], we also immunized animals with a licensed whole-virus H1N1pdm09 vaccine. Because of previous reports that the HI assay using chicken erythrocytes is insensitive for the detection of antibodies induced by H7, but can be improved by using horse erythrocytes [23,24], HI assays were done using erythrocytes from both species.

Immunization with the H7N9 vaccine induced dose-dependent antibody responses in guinea pigs, as measured by HI, MN and NAi assays (Table 2, upper panel). The dose-dependency of the antibody responses, irrespective of assay, was highly statistically significant (p<0.0001). All (100%) of guinea pigs receiving the 3.75 μg dose seroconverted in all antibody evaluations, with geometric mean titers (GMTs) of 3880, 1280, 3620 and 747 for HI (horse erythrocytes), HI (chicken erythrocytes) MN and NAi antibodies, respectively. Assessment of HI antibody titers using horse erythrocytes in the HI assay resulted in consistently higher titers than using chicken erythrocytes; differences were significant (p<0.05) for the 0.75 μg and 0.15 μg doses. ED_50_ values for the individual serological assays were calculated as 16 ng for HI (horse erythrocytes), 16 ng for HI (chicken erythrocytes), 19 ng for MN, and 35 ng for NAi.

**Table 2:** HA and NA antibody responses induced by H7N9 and H1N1pdm09 vaccines in guinea pigs.

	HI (chicken erythrocytes)^a^		HI (horse erythrocytes)^a^		CPE-MN^a^		NAi^a^	
Dose(μg HA)	GMT^b^ (95% CI)	%SC^c^	GMT^b^ (95% CI)	%SC^c^	GMT^b^ (95% CI)	%SC^c^	GMT^b^ (95% CI)	%SC^c^
H7N9								
3.75	1280 (802–2043)	100	3880 (3004–5012)	100	3620 (2788–4702)	100	747 (394–1416)	100
0.75	557 (259–1201)	100	2079 (1170–3695)	100	1372 (669–2814)	100	86 (34–215)	80
0.15	92 (27–308)	90	343 (87–1352)	90	172 (39–758)	70	57 (24–133)	70
0.03	43 (18–104)	50	113 (42–307)	90	57 (20–158)	80	15 (11–21)	10
0.006	5 (5–6)	0	6 (4–10)	10	6 (4–10)	10	11 (9–13)	0
0.001	5 (5–5)	0	5 (5–5)	0	5 (5–5)	0	10 (10–10)	0
Buffer	5 (5–5)	0	5 (5–5)	0	5 (5–5)	0	10 (10–10)	0
ED_50_	16 ng (7–32)		16 ng (7–32)		19 ng (9–39)		35 ng (15–82)	
H1N1pdm09								
3.75	941 (515–1718)	100	n.d.	n.d.	1613 (797–3263)	100	4389 (2815–6842)	100
0.75	557 (202–1535)	90	n.d.	n.d.	735 (247–2189)	90	686 (206–2282)	100
0.15	121 (50–293)	90	n.d.	n.d.	86 (30–247)	70	53 (18–155)	50
0.03	10 (5–21)	20	n.d.	n.d.	11 (5–26)	30	16 (10–27)	20
0.006	7 (4–12)	10	n.d.	n.d.	6 (4–10)	10	13 (10–17)	0
0.001	5 (5–5)	0	n.d.	n.d.	5 (5–5)	0	12 (10–16)	0
Buffer	5 (5–5)	0	n.d.	n.d.	5 (5–5)	0	10 (10–10)	0
ED_50_	30 ng (15–62)		n.a.		30 ng (15–62)		13 ng (3–41)	

^a^10 animals per dose group for both vaccines.

^b^Geometric mean titer

^c^Seroconversion (≥4-fold increase in antibody titer and antibody titer ≥ 1:40)

n.d., not done; n.a., not applicable

Antibody responses induced by the H7N9 whole-virus vaccines in mice are shown in Table 3 (upper panel). In agreement with the guinea pig data, immunization of mice with the H7N9 vaccine induced highly significant (p<0.0001) dose-dependent antibody responses as measured by HI, MN and NAi assays. In animals receiving the 3.75 μg dose, 90–100% seroconverted in all antibody evaluations, with GMTs of 905, 155, 520 and 226 for the HI (horse erythrocytes), HI (chicken erythrocytes), MN and NAi antibodies, respectively. ED_50_ values for the individual serological assays were calculated as 80 ng for HI (horse erythrocytes, 330 ng for HI (chicken erythrocytes), 169 ng for MN, and 401 ng for NAi. Assessment of H7N9 vaccine-induced HI antibody titers using horse erythrocytes in the HI assay resulted in consistently higher titers than using chicken erythrocytes; differences were highly significant (p<0.001) for the three highest doses.

**Table 3:** HA and NA antibody responses induced by H7N9 and H1N1pdm09 vaccines in DBA/2J mice.

	HI Chicken^a^		HI Horse^b^		CPE-MN^c^		NAi^b^	
Dose(μg HA)	GMT^d^ (95% CI)	%SC^e^	GMT^d^ (95% CI)	%SC^e^	GMT^d^ (95% CI)	%SC^e^	GMT^d^ (95% CI)	%SC^e^
H7N9								
3.75	155 (97–246)	90	905 (293–2793)	100	520 (345–783)	100	226 (79–648)	90
0.75	29 (19–45)	55	139 (40–488)	70	117 (58–236)	80	57 (20–162)	60
0.15	15 (11–23)	16	80 (31–209)	78	32 (16–63)	42	16 (9–29)	30
0.03	11 (9–13)	5	15 (6–36)	11	11 (9–13)	5	10 (10–10)	0
0.006	10 (10–10)	0	10 (10–10)	0	10 (10–10)	0	10 (10–10)	0
0.001	10 (10–10)	0	10 (10–10)	0	10 (10–10)	0	10 (10–10)	0
Buffer	10 (10–10)	0	10 (10–10)	0	10 (10–10)	0	10 (10–10)	0
ED_50_	330 ng (193–570)		80 ng (36–173)		169 ng (102–279)		401 ng (177–970)	
H1N1pdm09								
3.75	355 (182–692)	95	n.d.	n.d.	184 (59–575)	90	172 (63–468)	90
0.75	178 (76–416)	85	n.d.	n.d.	61 (14–257)	70	40 (12–129)	50
0.15	37 (16–85)	40	n.d.	n.d.	12 (10–16)	30	23 (11–50)	30
0.03	10 (10–10)	0	n.d.	n.d.	10 (10–10)	0	10 (10–10)	0
0.006	10 (10–10)	0	n.d.	n.d.	10 (10–10)	0	10 (10–10)	0
0.001	10 (10–10)	0	n.d.	n.d.	10 (10–10)	0	10 (10–10)	0
Buffer	10 (10–10)	0	n.d.	n.d.	10 (10–10)	0	10 (10–10)	0
ED_50_	150 ng (100–226)		n.a.		401 ng (177–970)		347 ng (144–922)	

^a^20 animals per dose group for both vaccines.

^b^10 animals per dose group for both vaccines.

^c^20 animals per dose group for H7N9 vaccine; 10 animals per dose group for H1N1 pdm09 vaccine.

^d^Geometric mean titer

^e^Seroconversion (≥4-fold increase in antibody titer and antibody titer ≥ 1:40)

n.d., not done; n.a., not applicable.

There was a highly significant correlation of MN titers with HI titers in guinea pigs and mice for HI assays utilizing horse erythrocytes (r = 0.97, p<0.0001 and r = 0.94, p<0.0001, respectively) and chicken erythrocytes (r = 0.97, p<0.0001 and r = 0.84, p<0.0001, respectively). A highly significant correlation (r≥0.84, p<0.0001) between vaccine-induced NAi titers and HI or MN titers was also calculated in both species, indicating that immune responses directed against both the HA and NA proteins of the whole-virus H7N9 vaccine increase proportionally in a dose-dependent manner.

Antibody responses induced by the H1N1pdm09 vaccine in guinea pigs and mice are shown in Table 2 (lower panel) and Table 3 (lower panel), respectively. Comparison of antibody responses induced by the H7N9 and H1N1pdm09 vaccines shows that HI, MN and NAi antibody titers and seroconversion rates were very similar for the H7N9 and H1N1pdm09 vaccines in both animal models. HI titers induced by the H7N9 and H1N1pdm09 vaccines in guinea pigs were statistically equivalent (p>0.05) irrespective of the source of erythrocytes utilized in the HI assay. In mice, when HI assays were done utilizing chicken erythrocytes, lower HI antibody titers were induced by the H7N9 vaccine compared to the H1N1pdm09 vaccine, when comparing the 0.75 μg doses (p<0.001) and the 0.15 μg doses (p<0.05), but titers were equivalent at other doses (p>0.05). Moreover, when horse erythrocytes were utilized in the H7N9 HI assay, HI titers induced by the H7N9 and H1N1pdm09 vaccines were equivalent (p>0.05) at all doses. MN titers induced in mice and guinea pigs by the H7N9 and H1N1pdm09 vaccines were equivalent (p>0.05) at all doses. NAi titers induced by the H1N1pdm09 vaccine in guinea pigs were higher for the two highest antigen doses (p<0.001) but equivalent at all other doses (p>0.05). In mice, NAi titers induced by the H7N9 and H1N1pdm09 vaccines were equivalent (p>0.05) at all doses. Importantly, irrespective of animal model or serological assay, ED_50_ values calculated for the H7N9 and H1N1pdm09 vaccines were very similar, with overlapping 95% CI values for all comparisons. Taken together, these data indicate that the whole-virus H7N9 and H1N1pdm09 vaccines are similarly immunogenic in mice and guinea pigs.

### Dose-dependent protection of immunized mice against challenge with H7N9 virus

**Fig 1:**
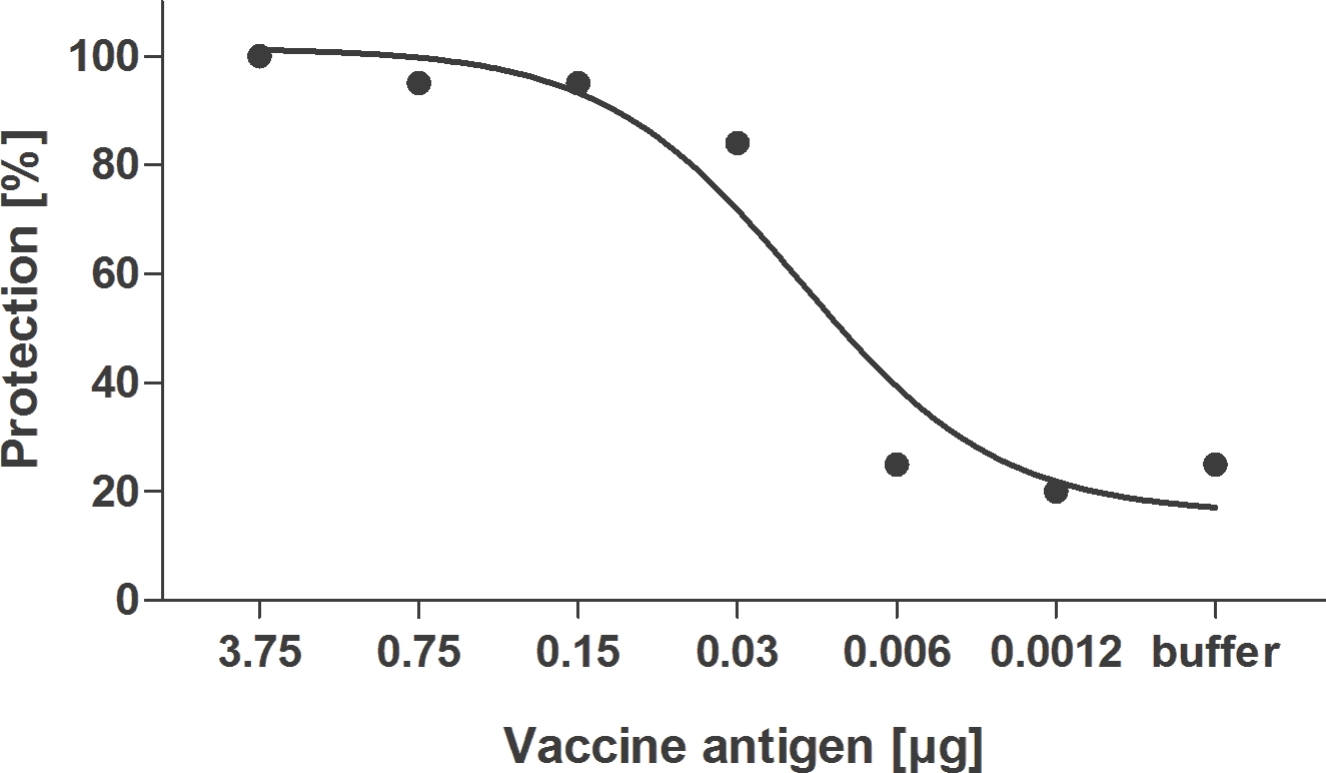
Dose-dependent survival of DBA/2J mice immunized with whole-virus H7N9 vaccine against lethal challenge with wild-type H7N9 virus.

**Fig 2:**
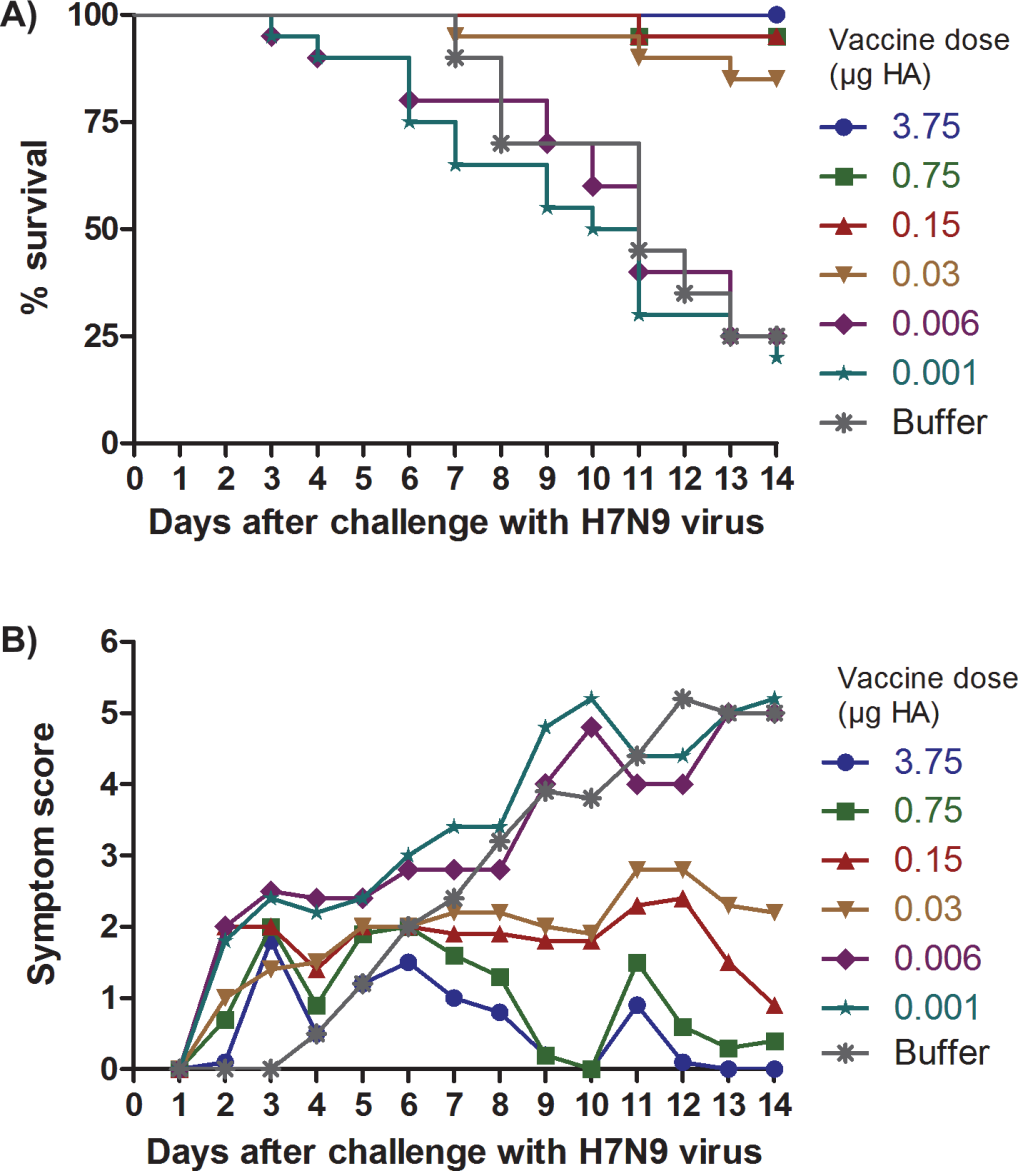
Dose-dependent protection against disease symptoms and death in H7N9-challenged DBA/2J mice immunized with whole-virus H7N9 vaccine. (A) Kaplan-Meier survival curves, (B) Severity and duration of disease symptoms

Pilot challenge experiments (data not shown) using CD1, BALB/c, and DBA/2J mice, as well as guinea pigs indicated that only DBA/2 mice exhibited visible disease symptoms and death after challenge with H7N9 virus (LD_50_ = 20 TCID_50_). No consistent symptoms or death were seen in any of these models after challenge with H1N1pdm09, so no challenge studies were done with that virus. Consequently, the ability of the whole-virus H7N9 vaccine to protect against high titer lethal H7N9 virus challenge was assessed in immunized DBA/2J mice. Mice which had received two immunizations, three weeks apart, were challenged with high titer (1 x 10^5^ TCID_50_; 5000 LD_50_) of wild-type H7N9 virus. Fig. 1 shows that the whole-virus H7N9 vaccine provides dose-dependent protection against lethal challenge with H7N9 virus. 100% of animals receiving the 3.75 μg vaccine dose survived lethal challenge, compared to only 25% of animals receiving buffer control (Fig. 1, Fig. 2A). Even at a very low dose of 0.03 μg, 84% of immunized animals survived H7N9 virus challenge, although a clear dose-dependent reduction in disease symptoms was observed for vaccine doses between 3.75 and 0.03 μg (Fig. 2B). At vaccine doses lower than 0.03 μg, the proportion of animals surviving after 14 days was not increased compared to buffer control, and these lower doses were also not sufficient to prolong survival or reduce the severity of disease symptoms (Fig. 2). Survival of animals in the different dose groups was highly correlated with dose (r = 0.91, p<0.0001), as well as HI and MN antibody GMTs (r≥0.90, p<0.0001) and seroconversion rates (r≥0.89, p<0.0001). The role of antibodies in protection was also supported by the fact that only one mouse with an HI titer of ≥1:40 succumbed to lethal challenge. This mouse was also seronegative in both MN and NAi assays. All other mice which achieved HI seroconversion were protected. The PD_50_ value for the H7N9 vaccine was calculated to be 0.008 to 0.01 μg HA.

### T-helper cell and IgG subclass responses in immunized mice

**Fig 3:**
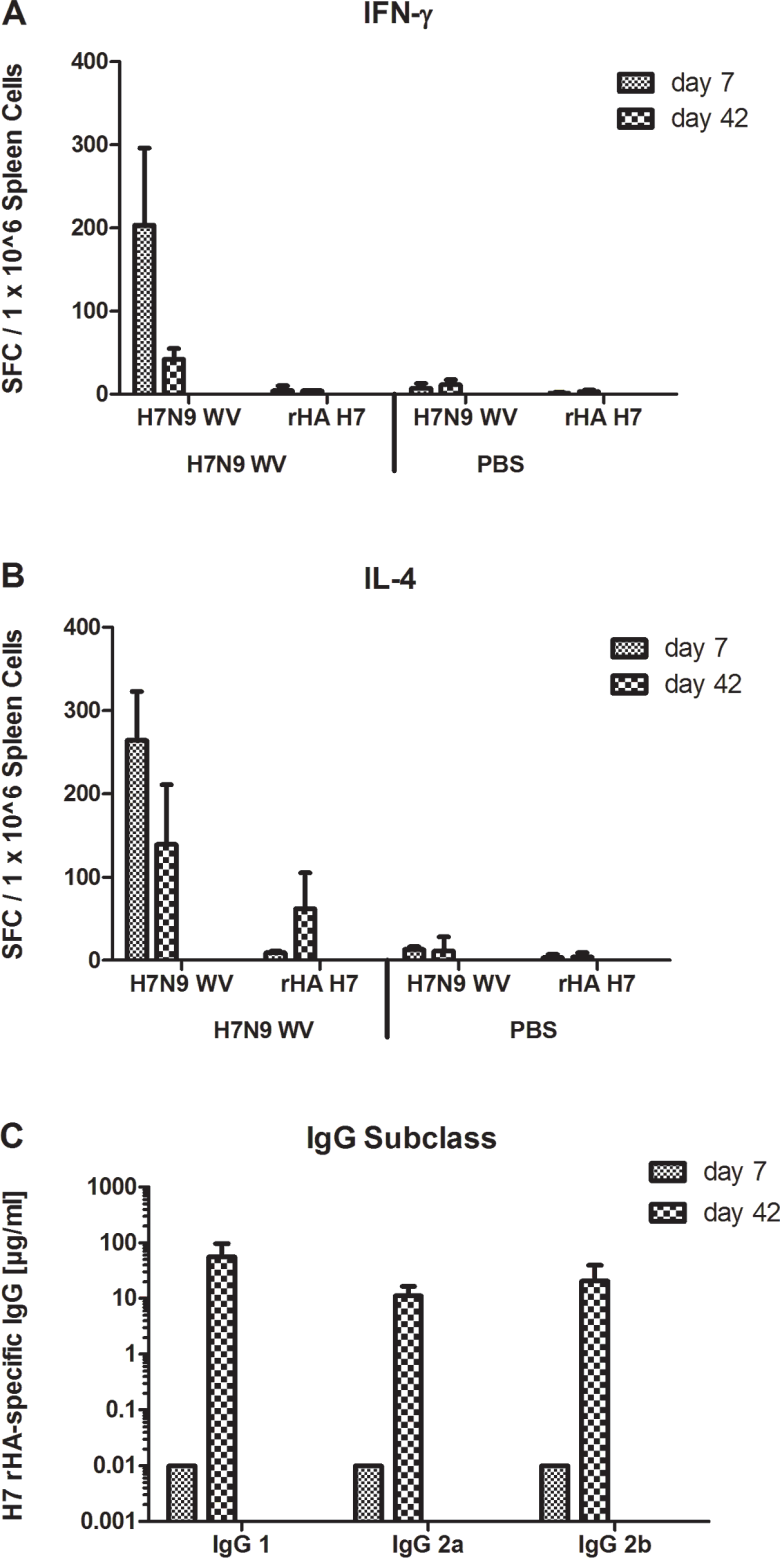
Th-1 and Th-2 cytokine and IgG subclass responses in mice immunized with pandemic H7N9. DBA/2J mice were immunized with whole-virus (WV) H7N9 vaccine or buffer as control on days 0 and 21. Spleen cells were collected 7 days after the first, or 21 days after the booster immunization (i.e. 42 days after the first), and stimulated with whole-virus (WV) H7N9 vaccine or recombinant H7 HA (rHA) for the determination of cells responding to the antigens by secretion of either IFN-γ (A) or IL-4 (B) using an ELISPOT assay. Statistically significant differences of comparisons between T cell responses in mice receiving H7N9 vaccine or buffer are shown. H7 HA-specific IgG subclass responses were analyzed by ELISA using sera collected on day 42 (C). IgG subclass responses on day 42 were compared by ANOVA. ***, p<0.0001; ns, not significant.

To evaluate the type of T-helper (Th) cell responses induced by the whole-virus H7N9 vaccine, DBA/2J mice were immunized twice, three weeks apart, with the 3.75 μg HA vaccine dose. To distinguish between Th1- and Th2-type T cell responses, spleen cells from spleens harvested one week after the first immunization and three weeks after the second immunization were evaluated by IFN-γ (Fig. 3A) and IL-4 ELISPOT (Fig. 3B) analyses, respectively. To distinguish between T cell responses directed against HA and those against other influenza virus proteins, spleen cells were stimulated either with whole-virus H7N9 or recombinant HA (rHA). One week after the first immunization, a balanced Th1/Th2 type response was induced in immunized animals, with 200 to 300 spots per million spleen cells stimulated by whole-virus H7N9, in both the IFN-γ and IL-4 ELISPOT assays (p<0.0001 compared to animals receiving buffer). Three weeks after the second immunization, lower levels of both Th type responses were seen, but both Th1 and Th2 type responses remained substantially and significantly (p<0.0001) above those measured in control animals receiving buffer. Negligible IFN-γ responses were seen in spleen cells stimulated by rHA (p>0.05), and substantial IL-4 responses were only evident after the second immunization (p<0.0001), indicating that the majority of T-helper cell responses are likely directed against non-HA viral antigens.

To further substantiate the differences in the Th cell responses, immune sera collected at the same time points were evaluated by ELISA to distinguish between HA-specific IgG1, characteristic of a Th2 type response, and IgG2a and IgG2b responses, which are indicative of a Th1 type response (Fig. 3C). Three weeks after the second immunization, statistically equivalent (p>0.05) high titers of IgG1, IgG2a and IgG2b responses were evident, suggesting a mixed Th1/Th2 type response. Taken together, the T cell analyses and IgG subclass analyses demonstrate that the whole-virus H7N9 vaccine effectively induces vaccine-specific T cell responses which are characterized by a balanced Th1/Th2 type response.

## Discussion

A non-adjuvanted, Vero cell culture-derived whole-virus H7N9 vaccine was produced using identical methodologies previously used for the manufacture of licensed H1N1pdm09 and H5N1 vaccines [17,20]. Characterization of the H7N9 vaccine compared to the licensed H1N1pdm09 vaccine demonstrated that these vaccines have almost identical HA content and that the content of NA, NP and M1, are also highly similar in the two vaccines. The content of viral RNA in the H7N9 vaccine was substantially lower than in the H1N1pdm09 vaccine, despite the use of an identical method for vaccine preparation. However, this is in agreement with previous studies which demonstrated that viral RNA content can vary despite identical preparation methods (data not shown). Based on the highly similar antigenic identity of the whole-virus H7N9 and H1N1pdm09 vaccines, a comparative study was carried out to directly compare the immunogenicity and protective efficacy of these vaccines in small animal models.

Pilot experiments using CD1, BALB/c, and DBA/2J mice demonstrated that visible disease symptoms and death were seen only after challenge of DBA/2J mice with H7N9. This result is in contrast to previous studies which have demonstrated that 100% lethality could be achieved in BALB/c mice after challenge with a mouse-adapted A/Anhui/01/2013 H7N9 strain [25]. However, as we chose to use a wild-type human isolate without mouse adaptation in these studies, the DBA/2J model was used, based on the results of the comparison studies. The DBA/2J model has been used extensively in influenza virus studies and has been shown to be a suitable, well characterized model to evaluate influenza vaccines [26–30], although the mechanism of susceptibility to H7N9 infection may not be as well understood as in the BALB/c mouse model.

The whole-virus H7N9 vaccine was highly immunogenic in guinea pigs and mice and provided dose-dependent protection of mice against lethal challenge with wild-type H7N9 virus. Antibody responses induced by the H7N9 vaccine were very similar to those induced by a licensed whole-virus H1N1pdm09 vaccine.

High HI titers were induced by the H7N9 vaccine in both animal models as measured in HI assays using either chicken or horse erythrocytes. Previous studies had indicated that chicken erythrocytes are insensitive for the detection of H7 HA antibodies, as a result of the relatively low density of α2,3-linked sialic acid receptors on chicken compared to horse erythrocytes [23,24]. The ability of the H7N9 vaccine to induce HI antibodies which are detectable by HI assays using either horse or chicken erythrocytes might be due to the reported ability of the novel H7N9 virus to bind efficiently to both avian (α2,3-linked sialic acid) and human (α2,6-linked sialic acid) receptors [7–9]. Nevertheless, although high HI titers were detected with HI assays utilizing erythrocytes from both species, HI antibody titers were consistently higher when measured using a horse erythrocyte-based HI assay, indicating that horse erythrocytes are more sensitive than chicken erythrocytes for the detection of H7N9 antibodies. HI titers induced in mice and guinea pigs by the H7N9 and H1N1pdm09 vaccines were statistically equivalent when horse erythrocytes were used to measure H7N9 antibodies. Importantly, the specificity of the HI assay was not impacted by the use of horse erythrocytes, as evidenced by the fact that all sera from animals receiving buffer were negative.

Seasonal influenza vaccines are licensed on the basis of HI antibody titers, which have been shown to correlate with clinical protection [31,32]. However, there is no established correlate of protection against human infection with avian influenza viruses, and alternative correlates of protection, such as virus-neutralizing and NA-inhibiting antibodies, are also being investigated. Virus-neutralizing antibodies induced by an H5N1 vaccine in humans have been demonstrated to correlate with protection against virus challenge in passive transfer studies in mice [33], and virus-neutralizing antibody assessments have been used to support the licensure of H5N1 vaccines in Europe [34,35]. NA-specific antibodies have also been demonstrated to correlate with protection in animals and humans [36,37]. In the present study, both MN and NAi antibodies were effectively induced by the whole-virus H7N9 vaccine, at titers similar to those induced by the licensed whole-virus H1N1pdm09 vaccine. Moreover, there was a highly significant correlation between all HI, MN and NAi antibody responses, and between antibody responses and protection against lethal challenge. Interestingly, low doses of H7N9 vaccine, for example, 0.03 and 0.150 μg HA, were able to protect against a lethal challenge even when the majority of mice had an antibody titer was below the limit of detection (Table 1, Fig. 2A). However, mice immunized with these doses demonstrated higher levels of disease symptoms than did those immunized with higher doses, where seroconversion was induced in at least 70% of mice (Table 1, Fig. 2B). The protection of seronegative immunized mice against a lethal H7N9 challenge in this study is consistent with previous results obtained with active or passive immunization against H5N1 avian influenza [20, 33]. Previous studies on the correlation survival of passively immunized mice with neutralizing antibody titer following challenge with wild-type H5N1 virus resulted in calculated PD_50_ values of 1:5, that is, below the level of detection in the HI and MN assays [33].

Surprisingly, 100% lethality was not seen in the control challenge groups, despite the use of a 5000 LD_50_ challenge dose. The reason for this is not known. However, in previous studies, the use of a challenge dose of 10^5^ H5N1 also did not result in consistent death of all the buffer control mice [20]. It could be speculated that the high challenge dose of cell culture-derived virus used in these experiments could contain molecules (cytokines, chemokines, RNA) which could induce an antiviral state in the model animal, protecting some control animals from severe disease. This could also explain why some animals vaccinated with low doses developed symptoms faster than the buffer control. It is possible that the immunised mice receiving vaccine develop blocking antibodies to the partially protective Vero cell components. Buffer control animals which were not immunised with Vero cell material would not develop such antibodies and as such could be less susceptible to challenge virus.

In addition, the survival curves following challenge show an unusual pattern in that animals die continuously from Day 4 to Day 14, in contrast to data previously published for H7N9 in the BALB/c model [25]. This may be a reflection of the fact that a non-mouse-adapted strain was used for challenge in a DBA/2J mouse model. This mouse strain is also known to produce higher levels of antiviral factors, such as cytokines and chemokines than the BALB/c or B6 mice [26,38], which may contribute to this different survival pattern. In addition to the effective induction of H7 HA and H7 NA-specific antibody responses, the whole-virus H7N9 vaccine induced a balanced H7N9-specific Th1/Th2 T cell response in mice, as evidenced by data from IFN-γ and IL-4 ELISPOT and IgG subclass ELISA assays. These data are in agreement with previous studies demonstrating that whole-virus vaccines elicit more effective Th1-type immune responses than split or subunit vaccines [17,39,40]. However, it has been demonstrated that the genetic background of inbred mouse strains can influence the cytokine response to influenza [26]. Thus, it cannot be absolutely concluded that the Th1 responses reported here are driven by the vaccine alone. Nevertheless, these data are in agreement with previous studies in BALB/c and C57BL/6 mice that show that whole virus vaccines induce more effective Th1-type immune responses subunit vaccines [41]. Th1-type CD4^+^ T cell responses are important for the activation of CD8^+^ T cells, macrophages and natural killer cells, which have a direct role in prevention of severe disease and mortality [42], whereas Th2-type CD4^+^ T cell responses provide cognate help to B cells, a prerequisite for immunoglobulin switch, affinity maturation and the establishment of B-cell memory [43]. A balanced Th1/Th2 type response is thus associated with the effective induction of humoral as well as cellular immune responses.

Taken together, comparison of HI, MN and NAi antibody responses induced by the whole-virus H7N9 vaccine and the licensed whole-virus H1N1pdm09 vaccine indicate that both vaccines are similarly highly immunogenic. Moreover, the induction of a potent, balanced T cell response by the H7N9 vaccine in DBA/2 mice, as was demonstrated earlier for an H1N1pdm vaccine in BALB/c mice [17], provides further evidence of the immunogenicity of the whole-virus H7N9 vaccine. These data are reassuring because it had been speculated that H7N9 vaccines might be less immunogenic than other influenza vaccines [15]. In this respect, the conclusions of our study could be questioned on the basis that standardized reagents were not available to quantify the HA content of the H7N9 vaccine, i.e. the critical vaccine component for induction of HI and neutralizing antibody responses. However, densitometric SDS gel analysis demonstrated that both the H7N9 and H1N1pdm09 vaccines compared in the study contained highly similar amounts of HA and other viral proteins. The viral RNA content of the H1N1pdm09 vaccine was significantly higher than that of the H7N9 vaccine, emphasizing that, although RNA might have contributed to the immunogenicity of both the H7N9 and H1N1pdm09 vaccines, this potential adjuvant effect of the viral RNA did not play a role in over-estimating the immunogenicity of the H7N9 vaccine.

Limited data for other H7N9 vaccines have been reported to date. An H7N9 virus like particle (VLP) vaccine, either non-adjuvanted or adjuvanted with a novel saponin-based adjuvant formulation, was reported to induce HI and NAi antibodies in mice [25], but the induction of neutralizing antibodies or T cells was not reported. In clinical studies, VLP [44] and subunit H7N9 vaccines [45] were both reported to require novel adjuvants to induce adequate antibody responses against H7N9 virus. Recently, egg-derived, inactivated, whole virus H7N9 vaccines have been shown to induce a protective immune response in mice, either at high doses of 2 to 50μg HA without adjuvant [46] or by use of a novel squalene adjuvant or alum [47]. In addition, an adjuvanted MDCK cell-derived H7N9 whole virus vaccine was shown to protect mice against a lethal challenge [48]. A whole virus vaccine also protected ferrets against severe disease but not infection, after a single 15μg, 30μg, or 50μg HA immunization with a non adjuvanted vaccine [49].

The same Vero cell technology used to manufacture the whole-virus H7N9 vaccine has previously been used to manufacture licensed H1N1pdm09 [16–18,50] and H5N1 vaccines [34,35, 51–54,]. For these vaccines, immunogenicity in non-clinical studies was highly predictive of clinical immunogenicity, as demonstrated in clinical studies for H5N1 [51–54] and H1N1pdm09 [16,18] vaccines, as well as in large-scale use in the field for the whole-virus H1N1pdm09 vaccine [19]. The effective induction of antibody and T cell responses and protective efficacy in mice thus indicates that the whole-virus H7N9 vaccine could provide an effective intervention against H7N9 if a human vaccine is needed in the future.
